# A Comprehensive Review of the Latest Approaches to Managing Hypercholesterolemia: A Comparative Analysis of Conventional and Novel Treatments: Part I

**DOI:** 10.3390/life15081185

**Published:** 2025-07-25

**Authors:** Ema-Teodora Nițu, Narcisa Jianu, Cristina Merlan, Darius Foica, Laura Sbârcea, Valentina Buda, Maria Suciu, Adelina Lombrea, Dana Emilia Movilă

**Affiliations:** 1 Faculty of Pharmacy, “Victor Babeș” University of Medicine and Pharmacy, 2 Eftimie Murgu Square, 300041 Timișoara, Romania; ema-teodora.nitu@umft.ro (E.-T.N.); narcisa.dinu@umft.ro (N.J.); cristina.merlan@umft.ro (C.M.); buda.valentina@umft.ro (V.B.); suciu.maria@umft.ro (M.S.); adelina.lombrea@umft.ro (A.L.); 2 Advanced Instrumental Screening Center, Faculty of Pharmacy, “Victor Babeş” University of Medicine and Pharmacy, 2 Eftimie Murgu Square, 300041 Timisoara, Romania; 3 Doctoral School, “Victor Babeş” University of Medicine and Pharmacy, 2 Eftimie Murgu Street, 300041 Timisoara, Romania; darius.foica@umft.ro; 4 Research Centre for Pharmaco-Toxicological Evaluation, “Victor Babeș” University of Medicine and Pharmacy, 2 Eftimie Murgu Square, 300041 Timisoara, Romania; 5 University Clinic of Internal Medicine and Ambulatory Care, Prevention and Cardiovascular Recovery, Department VI—Cardiology, “Victor Babeș” University of Medicine and Pharmacy, 300041 Timisoara, Romania; man.dana@umft.ro; 6 Research Centre of Timisoara Institute of Cardiovascular Diseases, “Victor Babeș” University of Medicine and Pharmacy, 300041 Timisoara, Romania

**Keywords:** hypercholesterolemia, lipid-lowering therapies, cardiovascular risk reduction, statins, ezetimibe, emerging therapies for dyslipidemia

## Abstract

Hypercholesterolemia is a major modifiable risk factor for atherosclerotic cardiovascular disease (ASCVD), affecting a significant proportion of the adult population worldwide. This narrative review provides a comprehensive and up-to-date overview of hyperlipidemia management, spanning from epidemiological trends and underlying pathophysiological mechanisms to the limitations of conventional therapies such as statins and ezetimibe. Particular emphasis is placed on cardiovascular risk assessment, current stratification tools, and international guideline-based interventions. The present paper, focusing primarily on the biological mechanisms of dyslipidemia and the clinical use of traditional lipid-lowering agents, serves as the first part of a two-part series, preceding a forthcoming review of novel pharmacological approaches. Our data synthesis is based on a structured literature search conducted across Google Scholar, PubMed, and Scopus, including studies published up to June 2025. The review also includes aspects related to non-pharmacological strategies, pharmacoeconomic considerations, and pharmacogenetic influences on treatment response. Ultimately, this work aims to equip clinicians with evidence-based, nuanced insights essential for optimizing lipid management and reducing cardiovascular risk, while setting the foundation for understanding how emerging therapies may overcome current therapeutic limitations.

## 1. Introduction

Over the past three decades, cardiovascular disease (CVD) has emerged as the leading global cause of disease burden, accounting for 93% prevalence, 54% of mortality, and 60% of disability-adjusted life years lost. Disparities in disease outcomes both within and between continents are significant, leading to annual costs of USD 147 billion in healthcare and USD 216 billion due to lost productivity [1,2]. CVD holds the position as the primary cause of death worldwide [3,4]. Hypercholesterolemia is a critical factor in the development of lipid plaques, particularly linked to high levels of low-density lipoprotein (LDL), which contribute to an increase risk of CVD, peripheral arterial disease (PAD), and cerebrovascular disease [5,6,7].

Familial hypercholesterolemia (FH) is an inherited genetic condition characterized by lifelong elevated levels of LDL cholesterol (LDL-C). Most cases of FH arise from autosomal co-dominant inheritance, with mutations primarily found in the LDL receptor (LDLR), proprotein convertase subtilisin/kexin type 9 (PCSK9), apolipoprotein B (ApoB), and LDL receptor adaptor protein (LDLRAP) genes. Patients with FH have a significantly higher risk of developing atherosclerotic cardiovascular disease (ASCVD), heart valve conditions, and related complications, such as cardiovascular mortality and aortic stenosis [8]. FH often goes undiagnosed until a cardiovascular event occurs. Prompt diagnosis and treatment to lower LDL-C levels are essential for reducing the risks associated with ASCVD in these patients. Managing FH typically involves intensive lipid-lowering therapy, which may begin in childhood, with statins being the first-line treatment. Other effective medications, such as mipomersen, ezetimibe, bempedoic acid, PCSK9 inhibitors, and lomitapide, may also be appropriate [8].

**Figure 1:**
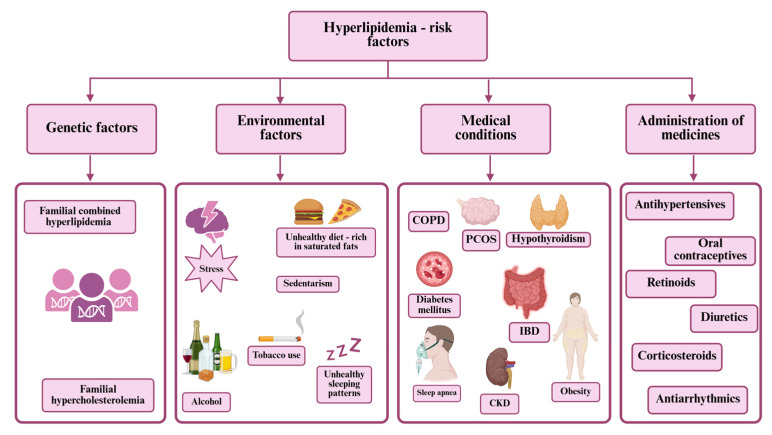
Risk factors of hyperlipidemia. Abbreviations: CKD—Chronic kidney disease; COPD—Chronic Obstructive Pulmonary Disease; IBD—Inflammatory Bowel Disease; PCOS—Polycystic ovary syndrome.

Research indicates that both the magnitude and duration of LDL-C exposure significantly contribute to the risk of ASCVD and its complications [9]. This underscores the importance of prioritizing and managing high LDL-C levels. Atherosclerosis is a complex vascular disease primarily characterized by the accumulation of lipid-laden plaques in the arterial intima [10]. According to the American Heart Association’s (AHA) 2025 Heart Disease and Stroke Statistical Update, 34% of U.S. adults—approximately 86 million people—have total serum cholesterol levels above 200 mg/dL. Furthermore, 3.65 million fatalities were linked to elevated LDL levels [11]. The Framingham Heart Study (FHS) demonstrated a strong correlation between increased total cholesterol and higher risk of ASCVD, suggesting that for every 10 mg/dL increase in total cholesterol, the risk rises by 5–10% [12]. Hyperlipidemia, indicated by elevated levels of ApoB, continues to be a major risk factor for ASCVD, alongside traditional risk factors (Figure 1) such as tobacco use, unhealthy lifestyles, and high blood pressure (BP) [13].

CVDs have a diverse nature and multiple causes, which lead to tailored treatment approaches for each individual patient. Traditional treatment methods are today complemented by technological advances in the field of ‘omics’—including genomics, metabolomics, epigenomics, proteomics, transcriptomics, and microbiomics. Taken together, these disciplines/fields contribute to a deeper understanding of disease mechanisms and to a clearer vision of the future of personalized medicine [14].

The growing body of genomic knowledge, combined with the lack of adequate clinical classification of genetic variants, presents a significant challenge for traditional medicine in treating both common and rare diseases. To overcome this issue and achieve personalized therapy, there must be a collaborative effort to functionally and clinically describe genomic variants. This can be accomplished through extensive global sequencing of the genomes of healthy individuals from diverse ethnic backgrounds [15]. Genomic research has revealed that polymorphisms affecting lipid metabolism can influence LDL levels and cardiovascular risk, highlighting the role of LDL in the pathogenesis of atherosclerosis. Additionally, data suggests that targeted therapies addressing these factors may offer potential cardiovascular benefits, especially when used in conjunction with statins. Clinical trial studies indicate that these new agents can lead to improved outcomes in managing atherosclerotic plaques and reducing cardiovascular events [7].

From a historical perspective, statins have been recognized as one of the most effective drugs for lowering cholesterol. More recently, non-statin agents have been introduced to treat hypercholesterolemia, offering a viable alternative with a promising cost–benefit ratio [5,16,17].

In modern cardiology, most diagnostic criteria and therapeutic treatment methods rely on population-based studies, which often overlook the importance of individualized patient management [18]. To address this issue, a comprehensive analysis of phenotypes and ‘omics’ data could help in grouping patient clusters. This approach would reduce disparities while promoting patient-centered clinical care. Additionally, it would enhance the quality of life for patients and help minimize complications through the use of novel biomarkers, AI-assisted diagnostics, targeted therapies, and thorough long-term risk evaluations [19].

There is strong evidence that inequalities in cardiovascular health are widespread, affecting different population subgroups based on sex, race, and ethnicity. These disparities lead to negative effects on health outcomes and quality of life. To reduce inequities in the prevalence and treatment of CVD among minority populations, we need to intensify efforts focused on risk factor management, adherence to clinical guidelines, and therapeutic education. Improving education is especially important, given its significant impact on overall health and quality of life [20].

The main objective of this review is to deliver a thorough and up-to-date overview of current approaches to managing hypercholesterolemia, with particular emphasis on the underlying pathophysiological mechanisms of dyslipidemia, its associated complications, the clinical importance of cardiovascular risk assessment, and the interpretation of current guideline recommendations. Additionally, the present paper explores the comparative efficacy, safety, and pharmacoeconomic aspects of conventional lipid-lowering therapies, such as statins and ezetimibe. By integrating epidemiological evidence, clinical practice guidelines, and pharmacogenetic factors, this article aims to support clinicians in making informed, personalized treatment decisions. As the first part of a two-part series, this review sets the foundation for a subsequent article (Part II), which will focus on emerging lipid-lowering therapies—including PCSK9 inhibitors (alirocumab, evolocumab), small interfering RNA (siRNA) therapy (inclisiran), ATP citrate lyase (ACL) inhibitor (bempedoic acid), microsomal triglyceride transfer protein (MTP) inhibitor (lomitapide), and angiopoietin-like protein 3 (ANGPTL3) inhibitor (evinacumab)—and their role in addressing therapeutic gaps in high-risk patient populations.

## 2. Materials and Methods—Literature Search Strategy

This paper was meticulously designed as a narrative review, with the aim of synthesizing the latest scientific reports on managing hypercholesterolemia, including its therapeutic approaches. The literature search, conducted between January 2025 and April 2025, was comprehensive, and the manuscript was drafted in May 2025, ensuring the inclusion of the most up-to-date information.

The bibliographic sources were filtered out through a structured query of the PubMed, Google Scholar, and Scopus databases, using keywords including: “dyslipidemia”, “lipid-lowering therapies”, “cardiovascular disease”, “statins”, “ezetimibe”, “ESC guidelines”, “AHA guidelines”. Articles published in the last five years (2020–2025) were prioritized to reflect the timeliness of data and clinical relevance of information. Rationally, a limited number of older sources were merged when those sources served as essential works or clinical guidelines crucial for understanding therapeutic evolution.

The validated types of papers accepted encompass narrative reviews, systematic reviews, meta-analyses, and clinical studies examining therapeutic interventions for dyslipidemia, with an emphasis on new directions in research. Exclusively, articles published in English, with fully accessible content, which had been peer-reviewed, were eligible for inclusion. Studies with a solid methodological design and high clinical relevance were prioritized, ensuring that the information is directly applicable to clinical practice. Moreover, the present study incorporates the latest international clinical guidelines coordinated by leading organizations such as the European Society of Cardiology (ESC), the American Heart Association (AHA), and the National Institute for Health and Care Excellence (NICE), accompanied by reports addressing the role of pharmacogenetics in individualizing lipid-lowering therapy. These guidelines constitute internationally certified standards of practice and reflect expert consensus on therapeutic protocols based on the most solid evidence available. The integration of information in this narrative review allowed the correlation of data from the specialized literature with current/up-to-date recommendations in clinical practice, facilitating a more applicable and relevant understanding of therapeutic strategies in dyslipidemia.

Regarding the exclusion criteria, in vitro studies were eliminated because they do not realistically reflect the physiological complexity of the human body and therefore provide limited relevance for clinical practice. However, they remain valuable for understanding the molecular and pharmacological mechanisms of the analyzed substances. Furthermore, publications in languages other than English were excluded in order to maintain consistency and avoid any confusion. Abstracts without access to the full text were not integrated, as they do not allow researchers to fully evaluate the methodology and quality of the data presented. Editorials, letters to the editor, opinion pieces, and other materials that have not been subjected to a formal peer review process were also excluded due to their lack of convincing guarantees of scientific accuracy and validity of the results.

In order to improve the accuracy of the selection, the bibliographies were manually reviewed. This process involved cross-referencing the cited sources in the selected articles with the original publications to establish supplementary relevant sources that were possibly initially omitted. The information collected was subsequently categorized thematically to create a clear and rigorously documented overview that captures the overview of the current trends in dyslipidemia therapy and emerging directions in clinical research.

## 3. Lipid Metabolism and Atherosclerosis—An Integrated Pathophysiological Perspective

**Figure 2:**
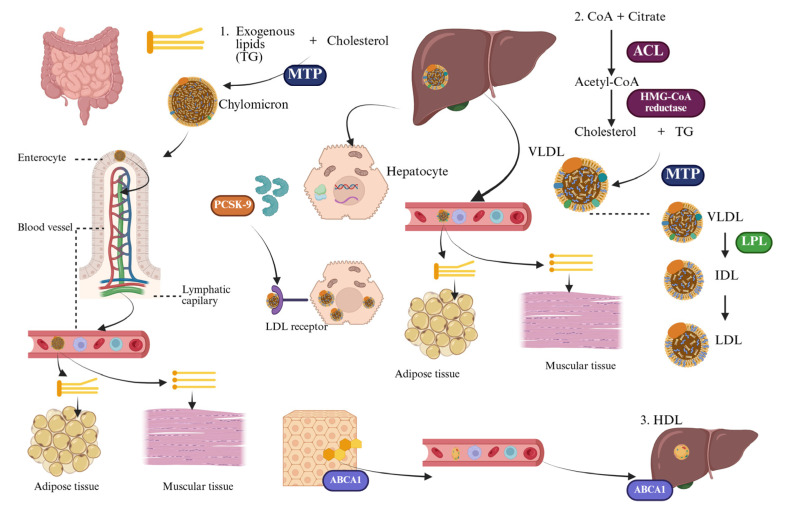
Lipid metabolism. 1. Transport of exogenous lipids via chylomicrons to peripheral tissues; 2. De novo synthesis of cholesterol. Transport of cholesterol via VLDL to peripheral tissues; 3. Transport of excess cholesterol via HDL back to the liver. Abbreviations: TG—Triglycerides; CoA—Coenzyme A; VLDL—Very low density lipoprotein; LDL—Low density lipoprotein; IDL—Intermediate density lipoprotein; HDL—High density lipoprotein; MTP—Microsomal triglyceride transfer protein; ACL—Adenosin triphosphate-citrate lyase; HMG-CoA reductase—Hydroxymethylglutaryl-CoA reductase; LPL- Lipoprotein lypase; PCSK9—Proprotein convertase subtilisin/kexin type 9; ABCA1—ATP-binding cassette transporter A1.

Lipid metabolism is essential to human physiology, playing a key role in maintaining membrane integrity, storing energy, synthesizing hormones, and regulating cellular signaling. Cholesterol, triglycerides (TG), phospholipids, and fatty acids are crucial to these processes. However, an excess or dysregulation of these lipids can turn them into pathogenic factors, promoting inflammation, endothelial dysfunction, and ultimately atherosclerosis [21]. Therefore, understanding lipid homeostasis and its associated disturbances is vital for uncovering the mechanisms behind cardiovascular disease and for supporting the use of modern lipid-lowering therapies (Figure 2).

Lipids are primarily synthesized in the liver, with additional production occurring in adipose tissue and the intestine. The process of de novo lipogenesis begins with cytosolic acetyl-CoA, which is mainly derived from mitochondrial citrate. Acetyl-CoA is converted to malonyl-CoA by the enzyme acetyl-CoA carboxylase, and this is further processed by fatty acid synthase to form palmitate. Cholesterol synthesis follows a different pathway that involves the rate-limiting enzyme HMG-CoA reductase. This enzyme catalyzes the conversion of HMG-CoA to mevalonate, a crucial step that is tightly regulated by cellular cholesterol levels [22]. In addition to being synthesized in the body, lipids are also obtained from dietary sources. The main classes of dietary lipids include TG, which are the principal energy source; phospholipids, which are essential components of cell membranes; and cholesterol, mainly derived from animal products. When consumed in excess—particularly saturated fats and trans fatty acids found in processed and high-fat animal products—these lipids can significantly affect plasma lipid levels and contribute to the development of dyslipidemia and related atherogenic processes [21].

Due to their hydrophobic nature, lipids cannot circulate freely in plasma and are transported as lipoproteins—spherical macromolecular assemblies composed of a hydrophobic TG core and cholesteryl esters, surrounded by a superficial monolayer formed by phospholipids, free cholesterol, and apolipoproteins [22]. These lipoproteins are classified based on their density and electrophoretic mobility into chylomicrons, very low-density lipoproteins (VLDL), intermediate-density lipoproteins (IDL), LDL, and high-density lipoprotein (HDL). Each class exhibits specific structural and metabolic roles, determined mainly by its apolipoprotein content [23].

LDL is the primary transporter of cholesterol, carrying it from the liver to peripheral tissues. Each LDL particle contains one molecule of apolipoprotein B-100, which acts as a ligand for LDLR, expressed mainly on hepatocytes. The LDL-LDLR complex is taken up (internalized) by cells through a process called receptor-mediated endocytosis, which allows for the delivery of cholesterol inside the cell. Under physiological conditions, this mechanism ensures the necessary supply of cholesterol for cell membrane synthesis and steroid production/steroidogenesis. However, when there are excessive LDL particles or when they undergo oxidative changes (known as oxidized LDL or oxLDL), they lose their ability to bind to LDLR. Instead, they attach to scavenger receptors on macrophages, leading to the formation of foam cells and the initiation of the atherosclerotic process [21].

In contrast, HDL plays a protective role against atherosclerosis by facilitating reverse cholesterol transport. This process involves the removal of excess cholesterol from peripheral cells, including macrophages found in the arterial intima, and transferring it back to the liver for excretion in bile. HDL particles, particularly those abundant in apolipoprotein A-I, engage with ATP-binding cassette (ABC) transporters, specifically ABCA1 and ABCG1, as well as the scavenger receptor class B type I (SR-BI), to promote cholesterol efflux. In addition to its role in lipid transport, HDL also has anti-inflammatory, antioxidant, and endothelial-stabilizing properties, which help counteract the harmful effects of LDL [22,24].

Disruptions in lipid metabolism often lead to clinical manifestations known as dyslipidemia, which is characterized by persistent abnormalities in the composition or concentration of circulating lipoproteins. Typically, these usually include elevated levels of LDL-C and TG, often paired with a decrease in high-density lipoprotein cholesterol (HDL-C). Dyslipidemias can be classified into various phenotypes, such as hypercholesterolemia, hypertriglyceridemia, mixed dyslipidemia, and isolated low HDL-C, each of which carries distinct implications for cardiometabolic health [21]. Importantly, dyslipidemia is not merely a biochemical anomaly; it is a critical and modifiable factor in the development of ASCVD. Prolonged exposure to excess atherogenic lipoproteins, especially small dense LDL particles, triggers endothelial activation, leading to a series of vascular events that promote oxidative stress, chronic low-grade inflammation, and progressive endothelial dysfunction—key processes in the development of atherosclerosis [25].

It is within this pathophysiological context that atherosclerosis emerges as the principal vascular complication of dyslipidemia. Although lipid imbalances may not show symptoms in their initial stages, their cumulative effects can damage arterial integrity, activate the immune system, and increase oxidative stress. This combination creates conditions that promote the formation of atheromatous plaques. Therefore, dyslipidemia serves not only as a marker of metabolic imbalance but also as a direct cause of the progression of arterial disease [26].

Atherosclerosis is primarily an inflammatory disease that affects the arterial wall, and it begins with endothelial dysfunction. Hemodynamic disturbances, such as low or oscillating shear stress—especially at arterial bifurcations—lead to increased permeability of the endothelium, allowing LDL particles to enter the subendothelial space. Once these lipoproteins are retained, they undergo oxidative modification, which triggers a series of events, including the adhesion of monocytes, their movement across the endothelium, and their differentiation into macrophages. These macrophages then internalize oxidized LDL (oxLDL) through scavenger receptors, transforming into foam cells, the earliest histological indication of atherosclerotic plaque [24].

As the disease advances, activated macrophages and smooth muscle cells release pro-inflammatory cytokines, reactive oxygen species, and matrix metalloproteinases. These substances promote further lipid accumulation, extracellular matrix remodeling, and the formation of a fibrous cap. An atherosclerotic plaque can remain stable for many years. However, under the influence of chronic inflammation and mechanical stress, the plaque can rupture, spilling prothrombotic materials into the bloodstream and triggering acute cardiovascular events such as myocardial infarction (MI) and ischemic stroke [21,24,27]. It is important to note that, in most cases, the plaques that rupture are not necessarily the most stenotic (narrowed) but rather the most inflamed and structurally vulnerable. The clinical consequences of advanced atherosclerosis can include potentially life-threatening events such as acute coronary syndromes, ischemic strokes, PAD, and sudden cardiac death. These complications result from progressive narrowing of the lumen, plaque instability, and thrombotic occlusion of critical arteries [27,28].

The protein PCSK9 plays a crucial role in regulating LDLR expression after it is synthesized. PCSK9 is mainly secreted by hepatocytes. When PCSK9 binds to LDLR on the surface of these cells, it triggers the internalization of the LDLR-PCSK9 complex through a process called endocytosis, leading to its degradation in lysosomes. This mechanism reduces the number of functional LDLRs on the surface of hepatocytes, resulting in higher levels of LDL-C in the bloodstream. Gain-of-function mutations in PCSK9 are linked to autosomal dominant hypercholesterolemia, while loss-of-function variants are associated with lower cholesterol levels and a decreased risk of cardiovascular events [29,30]. Recent data indicate that PCSK9 may play a role in regulating intracellular lipid processes, particularly through mechanisms like lipophagy, which is the autophagic degradation of lipid droplets in hepatocytes. This suggests a connection between the regulation of membrane receptor expression and cellular lipid breakdown. Furthermore, PCSK9 expression seems to be affected by inflammation and metabolic stress, highlighting its broader role in vascular biology beyond merely regulating cholesterol metabolism (Figure 2) [29,31].

Elevated LDL-C plays a crucial role in the development and progression of atherosclerosis, making genetically determined lipid imbalances particularly concerning from a clinical perspective. A key example of this is FH, a hereditary condition that significantly increases the risk of atherosclerosis, often starting in childhood [32]. FH impairs the clearance of LDL-C due to defects related to the LDLR, resulting in a lifelong elevated atherogenic potential. Early detection and intervention are essential to prevent premature cardiovascular issues and mortality. The most common cause of FH is mutations in the LDLR gene, which disrupt the normal functioning of the LDLR. Less frequently, mutations can occur in the ApoB gene, which affects the binding of LDL particles to their receptor, or in the PCSK9 gene, which increases the degradation of LDLR [33,34]. Homozygous FH can lead to plasma LDL-C levels exceeding 500 mg/dL and is associated with severe coronary artery disease (CAD) as early as childhood or young adulthood. Even in its heterozygous form, FH increases cardiovascular risk by 10 to 20 times compared to the general population if left untreated [35].

Given the genetic heterogeneity and variable clinical severity of FH, it is essential to distinguish between its two main phenotypic presentations: heterozygous FH (HeFH) and homozygous FH (HoFH). While both forms share a common pathogenic mechanism involving impaired clearance of LDL particles, they differ substantially in terms of LDL-C levels, age of onset, clinical manifestations, and response to lipid-lowering therapies. A clear understanding of these differences is crucial for accurate diagnosis, risk stratification, and personalized management [36].

HeFH is far more prevalent, with an estimated global frequency of approximately 1 in 250 individuals. It results from a single pathogenic variant in genes involved in LDLR function, most commonly LDLR, but also ApoB or PCSK9. Patients with HeFH typically exhibit a two- to three-fold increase in plasma LDL-C levels, often ranging between 190 and 400 mg/dL, and may remain asymptomatic until early adulthood. Clinical manifestations such as tendon xanthomas or early-onset CAD generally emerge in the third or fourth decade of life. HeFH is usually responsive to conventional lipid-lowering therapies, including statins, ezetimibe, and PCSK9 monoclonal antibodies, with most patients achieving meaningful reductions in LDL-C levels when treatment is initiated early and adhered to consistently (Table 1) [36,37].

In contrast, HoFH is a rare and far more severe condition, affecting approximately 1 in 300,000 individuals. It arises from biallelic pathogenic mutations in the same genes, either identical (true homozygotes) or different (compound heterozygotes), leading to a near-complete or total loss of LDLR activity. As a result, patients with HoFH often present extremely elevated LDL-C levels exceeding 500 mg/dL, with manifestations such as cutaneous xanthomas, arcus cornealis, and aortic stenosis occurring as early as childhood. If untreated, these individuals may experience MI or other major cardiovascular events before the age of 20. Standard lipid-lowering therapies that rely on residual LDLR function are often insufficient in HoFH, particularly in patients with two null LDLR mutations [38,39].

A key therapeutic distinction between the two forms lies in their response to receptor-dependent therapies. While HeFH patients typically retain enough receptor functionality to respond favorably to statins and PCSK9 inhibitors, HoFH patients, especially those with receptor-negative genotypes, may show minimal to no benefit from such treatments. This differential response underlines the importance of genetic testing not only for diagnosis but also for guiding therapeutic decision-making, especially in pediatric cases and those with an unusually severe lipid phenotype (Table 1) [36,39].

Briefly, both HeFH and HoFH result from mutations affecting the LDLR pathway, yet they differ markedly in clinical expression and therapeutic implications. Early recognition of HoFH is critical due to its aggressive course and the need for intensive treatment. Conversely, early identification and intervention in HeFH can markedly reduce long-term cardiovascular risk, especially when personalized therapy is guided by genetic and biochemical profiling [36,39].

In conclusion, the landscape of lipid metabolism is marked by a delicate balance between synthesis, transport, uptake, storage, and elimination. The contrasting roles of LDL-C and HDL-C, along with receptor-mediated regulation and the influence of PCSK9, highlight the complexity of this integrated system. Disruption at any stage can result in harmful lipid accumulation, vascular inflammation, and the clinical consequences of atherosclerosis. Therefore, therapeutic strategies targeting these mechanisms, including statins, ezetimibe, PCSK9 inhibitors, and novel lipid-lowering agents, should be considered not simply as lipid-lowering interventions but as specific approaches grounded in a deep and integrated understanding of human pathophysiology [21].

## 4. Complications of Hyperlipidemia

Hyperlipidemia, characterized by elevated levels of total cholesterol, LDL-C, TG, or atherogenic lipid fractions, is a major metabolic abnormality implicated in a broad spectrum of chronic diseases. While it often remains clinically silent in its early stages, persistent lipid dysregulation contributes to the pathogenesis of numerous complications across multiple organ systems. These include, but are not limited to, ASCVD, cerebrovascular events, chronic kidney disease (CKD), pancreatitis, and metabolic dysfunctions. Understanding the systemic consequences of hyperlipidemia is critical for guiding early detection, risk stratification, and the implementation of effective preventive and therapeutic strategies [40,41,42].

Hyperlipidemia is a key etiological factor in ASCVD, a condition characterized by the progressive accumulation of atheromatous plaques in arterial walls. Elevated LDL-C facilitates the infiltration of cholesterol particles into the subendothelial space, where oxidative modification triggers local inflammation and recruitment of macrophages. Foam cell formation and subsequent fibrous cap development are hallmarks of plaque maturation. Over time, these plaques may rupture, leading to acute thrombosis and vessel occlusion. Clinically, ASCVD manifests as CAD, cerebrovascular disease, and PAD. The risk associated with hyperlipidemia is cumulative and directly proportional to the magnitude and duration of LDL-C elevation. Lifelong exposure to high cholesterol significantly accelerates atherosclerosis, particularly in individuals with FH. Multiple studies, including those underpinning the ESC and the American Heart Association/American College of Cardiology (AHA/ACC) guidelines, have demonstrated that LDL-C reduction, especially through statins and PCSK9 inhibitors, leads to substantial decreases in major adverse cardiovascular events [21,43,44].

CAD is one of the most prevalent and devastating outcomes of dyslipidemia. It arises from the narrowing of coronary arteries due to lipid-laden plaques that compromise myocardial perfusion. Hyperlipidemia is particularly damaging in the context of other cardiovascular risk factors (Figure 1) such as smoking, diabetes, and hypertension. The transition from stable CAD to acute coronary syndrome often involves the rupture or erosion of an unstable plaque, followed by platelet activation and thrombus formation. Elevated LDL-C levels correlate with increased plaque vulnerability, and post-mortem studies have consistently shown lipid-rich cores in culprit lesions of MI. From a therapeutic standpoint, intensive lipid lowering is critical for both primary and secondary prevention. Clinical trials, such as IMPROVE-IT and FOURIER, have confirmed that achieving very low LDL-C levels (<55 mg/dL) is associated with significant reductions in MI incidence [44,45,46].

The pathophysiological link between hyperlipidemia and ischemic stroke is primarily mediated through carotid atherosclerosis and intracranial arterial disease. Cholesterol-rich plaques may form in the carotid bifurcation or vertebrobasilar system, restricting cerebral blood flow or serving as embolic sources. Oxidized LDL promotes endothelial dysfunction in cerebral arteries, impairs nitric oxide bioavailability, and promotes thrombogenesis. Patients with elevated cholesterol levels, especially in the presence of hypertension or diabetes, are at markedly increased risk for ischemic cerebrovascular events [47,48,49]. Guidelines now recommend statin therapy for stroke prevention in patients with atherosclerotic disease, recognizing LDL-C as a modifiable contributor to stroke burden [50].

PAD refers to the obstruction of peripheral arteries, most commonly in the lower extremities, due to atherosclerotic plaque buildup. Hyperlipidemia contributes to PAD by promoting endothelial activation, vascular smooth muscle proliferation, and chronic inflammation. Clinically, PAD may present with intermittent claudication, rest pain, or critical limb ischemia. In advanced cases, it can lead to non-healing ulcers or limb loss. Importantly, PAD is a marker of systemic atherosclerosis and carries a high risk of concurrent coronary and cerebrovascular events. Effective management of PAD includes intensive lipid-lowering therapy. ESC guidelines classify PAD as a very high-risk condition, mandating LDL-C goals below 55 mg/dL. Despite this, underdiagnosis and undertreatment of PAD remain significant challenges [51,52].

While not a direct consequence, hyperlipidemia contributes to the pathogenesis of hypertension by inducing vascular remodeling and reducing arterial compliance [53]. Lipid accumulation within the arterial wall increases stiffness and impairs baroreceptor sensitivity, thereby raising systolic blood pressure. Furthermore, dyslipidemia can exacerbate the hypertensive state by promoting renal microvascular damage and impairing pressure natriuresis [54]. The combination of hypertension and hyperlipidemia exerts synergistic effects on the heart and kidneys, accelerating the onset of left ventricular hypertrophy and CKD. Emerging evidence suggests that lipid-lowering therapy may modestly reduce BP and improve vascular compliance, though this effect is secondary to its primary cardiovascular benefits [53].

In patients with type 2 diabetes mellitus, dyslipidemia is often characterized by elevated TG, low HDL-C, and small, dense LDL particles, a profile known as diabetic dyslipidemia [55]. This lipid pattern is particularly atherogenic and strongly associated with microvascular and macrovascular complications. Hyperlipidemia contributes to diabetic nephropathy through multiple mechanisms: glomerular endothelial injury, mesangial expansion, and tubulointerstitial fibrosis. Lipid-induced oxidative stress and pro-inflammatory cytokines release exacerbate albuminuria and accelerate renal function decline [56]. Guidelines from ESC and AHA/ACC now classify diabetic patients with organ damage as very high-risk, advocating aggressive LDL-C lowering to slow the progression of both cardiovascular and renal disease [57].

Emerging data suggest that hyperlipidemia may also contribute to neurovascular aging and cognitive decline [58]. Chronic exposure to elevated LDL-C is associated with white matter lesions, cerebral microinfarcts, and impaired neurovascular coupling. While the exact mechanisms remain under investigation, possible pathways include endothelial dysfunction, systemic inflammation, and an impairment of cerebral microcirculation. Observational studies have linked midlife dyslipidemia with increased risk of vascular dementia and Alzheimer’s disease [59,60,61,62]. Vascular health remains a key determinant of the brain aging process, and appropriate lipid management may play a preventive role in select populations [63,64].

Beyond atherosclerosis, hyperlipidemia fosters a state of chronic low-grade inflammation. Oxidized LDL is a potent immunogenic molecule that activates macrophages and endothelial cells, promoting the release of interleukins and adhesion molecules [65,66]. This inflammatory milieu not only accelerates plaque progression but also contributes to systemic effects, including insulin resistance, hepatic steatosis, and metabolic syndrome. Inflammatory pathways also play a role in plaque destabilization, making them therapeutic targets in secondary prevention [67,68].

Effective prevention and management of dyslipidemia-related complications require individualized treatment strategies that incorporate clearly defined lipid targets adapted to the underlying pathology and the patient’s overall cardiovascular risk profile. Current evidence-based guidelines, including those issued by the European Society of Cardiology and European Atherosclerosis Society (ESC/EAS) as well as the Kidney Disease Improving Global Outcomes (KDIGO) initiative, emphasize that the intensity of LDL-C reduction should be proportionate to the cumulative burden of risk factors, comorbidities, and established atherosclerotic disease. For example, patients with documented ASCVD, diabetes mellitus complicated by target organ damage, or advanced chronic kidney disease are categorized as very high risk. They are recommended to achieve LDL-C levels below 55 mg/dL, accompanied by at least a 50% reduction from baseline values. In contrast, individuals without manifest atherosclerosis but with moderate or low overall risk may be managed with more permissive targets.

Additionally, in certain clinical scenarios such as severe hypertriglyceridemia, therapeutic priorities shift to focus on triglyceride lowering to prevent pancreatitis. At the same time, LDL-C goals remain important in guiding long-term cardiovascular prevention strategies.

Table 2 summarizes the recommended lipid targets across the main pathologies discussed in this section, offering a structured approach to risk-adapted lipid management based on contemporary guideline recommendations [69,70].

Concluding, hyperlipidemia is a central pathophysiological factor linking lipid dysmetabolism, vascular injury, and inflammation. Its complications are diverse, clinically significant, and often synergistic, reinforcing the imperative for early and aggressive intervention guided by cardiovascular risk.

## 5. Cardiovascular Risk Assessment and Its Role in the Management of Dyslipidemia—Guidelines and Implications

CVDs remain the leading cause of death globally, with dyslipidemia recognized as a major modifiable risk factor [71]. Despite advancements in diagnostics and treatment, hyperlipidemia continues to significantly impact morbidity, mortality, and healthcare systems. Since lipid abnormalities contribute to the formation of atherosclerotic plaques, early identification and targeted management of dyslipidemia are essential to reduce the burden of cardiovascular disease [72].

Contemporary approaches to dyslipidemia have transitioned from fixed cholesterol targets toward comprehensive, risk-based strategies. This shift emphasizes global cardiovascular risk assessment to tailor the intensity and type of lipid-lowering therapy. Both ESC and AHA/ACC guidelines advocate for early and individualized intervention in patients with high or very high cardiovascular risk [17,73,74].

The rationale for this paradigm lies in the cumulative and synergistic effects of multiple risk factors (Figure 1) on cardiovascular outcomes. A moderately elevated LDL-C level in a patient with diabetes or a history of smoking may imply a significantly higher absolute risk than a higher LDL-C level in a patient without other comorbidities. Consequently, the estimation of absolute cardiovascular risk over a 10-year period has become the cornerstone of both primary and secondary prevention strategies [75].

Validated tools such as the Systematic Coronary Risk Evaluation 2 (SCORE2) (endorsed by the ESC) and the Pooled Cohort Equations (PCE, developed by ACC/AHA) guide clinicians in stratifying patients and optimizing treatment plans. SCORE2 estimates fatal and non-fatal events and incorporates country-specific recalibration, while PCE includes race, HDL-C, and diabetes status, allowing refinement of risk prediction in the U.S. population. Both tools allow integration of modifiers such as coronary artery calcium (CAC) scoring and other biomarkers, particularly in patients with intermediate risk (Table 3) [74].

While SCORE2 adopts a “treat-to-target” approach with precise LDL-C goals by risk category, the AHA/ACC guidelines focus on percentage reductions in LDL-C levels and shared decision-making (Table 4). Despite these operational differences, both frameworks converge on the principle of escalating treatment intensity in proportion to cardiovascular risk [74]. Effective implementation of these guidelines, including periodic reassessment and individualized planning, has been shown to improve clinical outcomes and optimize resource allocation. Continuous refinement of these models in diverse populations, including older adults and patients with comorbidities, remains an important focus of ongoing research [76,77,78].

Both the ESC and AHA/ACC guidelines recommend initiating high-intensity statin therapy (e.g., atorvastatin ≥ 40 mg, rosuvastatin ≥ 20 mg) as first-line treatment in high-risk individuals. When LDL-C goals or percentage reductions are not achieved, ezetimibe is typically added, followed by PCSK9 inhibitors in very high-risk cases. A central operational difference remains: ESC emphasizes absolute LDL-C targets, whereas AHA/ACC guidelines prioritize percentage-based reductions and threshold-driven intervention, such as adding non-statin therapy if LDL-C persists ≥ 70 mg/dL in secondary prevention. In contrast, the NICE guideline adopts a more conservative and cost-sensitive approach. NICE recommends high-intensity statins as the primary strategy, aiming for a ≥40% reduction in non-HDL cholesterol rather than fixed LDL-C thresholds. Ezetimibe is reserved for patients who do not achieve adequate lipid lowering or who are intolerant to statins, while PCSK9 inhibitors are strictly restricted to individuals with familial hypercholesterolemia or established cardiovascular disease who meet specific eligibility criteria. Despite robust recommendations across guidelines, implementation remains suboptimal due to factors such as therapeutic inertia (i.e., failure to escalate therapy when targets are unmet), limited access to advanced therapies, and patient non-adherence. Addressing these gaps requires clinical decision support tools, multidisciplinary collaboration, and enhanced patient education [74,77,79,80].

## 6. Treatment

### 6.1. Non-Pharmaceutical Approach

Most treatments typically start with lifestyle adjustments, which include adopting a healthy diet that is low in sodium, saturated fat, and alcohol, as well as engaging in regular physical activity instead of leading a sedentary lifestyle. In some cases, pharmacological therapy may also be necessary along with these lifestyle changes [81,82].

It is crucial to pay special attention to weight control, as it plays a vital role in preventing and reducing cardiovascular risks. This is especially important for obese individuals with a body mass index (BMI) of 30 kg/m^2^ or higher, as well as for those who are classified as overweight, with a BMI between 25 and 29.9 kg/m^2^, particularly if they have two or more additional risk factors [82]. For individuals with severe obesity or those who have co-morbidities, options such as bariatric surgery and medications like orlistat, bupropion, naltrexone, or high-dose glucagon-like peptide (GLP)-1 receptor agonists have shown significant efficacy in managing these conditions [4,83,84,85,86,87].

From a metabolic perspective, unhealthy weight involves an elevated visceral fat mass, lower leg fat mass, and decreased muscle mass. Visceral adipose tissue indicates the dominant burden of metabolically unhealthy obesity, while subcutaneous fat is metabolically inactive. Assessing waist circumference could more accurately reflect visceral adipose tissue, which is related to CVD risk, than BMI by itself. Therefore, people with comparable BMIs are able to endure diverse cardiometabolic risks. Concerning waist circumference, ESC guidelines recommend avoiding subsequent weight gain for values >88 cm in women and >94 cm in men [88].

An examination of the Mediterranean diet highlights its focus on fruits, vegetables, whole grains, olive oil, legumes, and nuts, with moderate consumption of red wine and fish. This combination offers significant benefits for heart health [73]. Healthy fats, particularly, play a crucial role in maintaining optimal lipid profiles and overall cardiovascular health. Fats are categorized based on their source and structure. Animal fats tend to be high in saturated fatty acids, whereas vegetable fats contain higher levels of unsaturated fatty acids, such as monounsaturated fats and polyunsaturated fats (PUFAs) (Table 5) [89]. Omega-3 and omega-6 PUFAs, particularly eicosapentaenoic acid and docosahexaenoic acid, can help lower TG by 10–50% and provide cardioprotective benefits. A recommended intake of 2–4 g per day can aid in reducing TG levels. On the other hand, trans fatty acids (Table 5), commonly found in full-fat dairy and processed foods, are known to increase LDL-C levels while reducing HDL-C levels, which leads to adverse lipid profiles [4]. In contrast, phytosterols can reduce LDL-C levels by 6–12% when consumed at a rate of 2 g per day, mainly by lowering cholesterol absorption [89].

Similarly, the Dietary Approaches to Stop Hypertension (DASH) diet, which emphasizes fruits, vegetables, and low-fat dairy products, effectively lowers BP and reduces cardiovascular risk, contributing to a 20% decrease in disease risk for those who adhere to it [4,93].

In patients with FH, dietary management plays a critical role in reducing LDL-cholesterol, maintaining a healthy weight, decreasing vascular inflammation and oxidative stress, and preventing additional risk factors such as diabetes and hypertension. It is recommended to strictly limit saturated fats (e.g., fatty red meat, butter, full-fat dairy), trans fats (e.g., industrial baked goods), dietary cholesterol (e.g., organ meats, excessive egg yolks), refined sugars, and ultra-processed foods, as these contribute significantly to elevated LDL-cholesterol and cardiovascular risk. Instead, the diet should emphasize the intake of unsaturated fats (such as olive oil, avocado, and fatty fish), soluble fiber (found in oats and legumes), fresh fruits and vegetables (at least five servings per day), whole grains, and functional foods enriched with plant sterols (~2 g/day), which have been shown to effectively lower LDL-cholesterol and improve lipid profiles [69,79].

### 6.2. Pharmaceutical Approach

#### 6.2.1. Statins

Statins are currently the primary therapeutic option for managing hypercholesterolemia and for the primary and secondary prevention of coronary and cardiovascular diseases. This class of medications includes fungal-derived statins such as lovastatin, which was the first statin introduced into therapy, as well as pravastatin and simvastatin, which are semi-synthetic. Additionally, there are synthetic statins, often referred to as “super statins,” which include fluvastatin, atorvastatin, rosuvastatin, and pitavastatin [94]. They work by competitively inhibiting the HMG-CoA reductase, which is a key regulator in cholesterol biosynthesis. This inhibition lowers cholesterol levels within hepatocytes. As a result, the LDLR expression, on the surface of hepatocytes, is increased. This enhances the uptake of LDL-C from the bloodstream, leading to a decrease in its circulating levels, as well as a reduction in the plasma concentration of other ApoB-containing lipoproteins, including TG [4,69,95,96]. In addition to lowering LDL-C and TG levels while increasing HDL-C levels, statins also exhibit pleiotropic effects, such as anti-inflammatory and antioxidant properties. They improve vascular endothelial function, inhibit the progression of atherosclerotic plaques, and reduce platelet aggregation, thereby exerting antithrombotic actions [69,96,97]. Statin use in patients with coronavirus COVID-19 reduced the risk of progression of disease severity and in-hospital death owing to anti-inflammatory, antithrombotic, and anti-oxidative properties of this therapeutic class, as well as inhibition of viral replication [98,99,100].

Statins have varying solubility profiles. Simvastatin, atorvastatin, fluvastatin, and lovastatin are relatively lipophilic, while pravastatin and rosuvastatin are more hydrophilic [94,101]. These characteristics significantly influence their absorption, bioavailability, plasma protein binding, and excretion. Hydrophilic statins primarily target hepatic tissues, resulting in a lower potential for affecting extrahepatic tissues, which are more susceptible to lipophilic statins due to passive diffusion. With the exception of pravastatin, statins undergo hepatic metabolism through cytochrome P450 (CYP450) isoenzymes, though pitavastatin and rosuvastatin are minimally affected by this process. This metabolic pathway can lead to potential pharmacokinetic interactions with other drugs that are also metabolized in the liver. Simvastatin and lovastatin are administered as prodrugs, whereas the other statins are used in their active forms. Additionally, lipophilicity contributes to the varying degrees of adverse effects associated with different statins [69,95,96,98].

The effectiveness of statins in lowering LDL-C levels depends on the dosage, the type of statin used, and individual patient differences. High-intensity statin therapy, which includes atorvastatin (at doses of 40 or 80 mg) and rosuvastatin (at doses of 20 or 40 mg), can achieve an LDL-C reduction of 50% or more. This significant reduction is associated with a decreased risk of major cardiovascular events. Moderate-intensity statins, such as lovastatin (40 mg), pravastatin (at doses of 40 or 80 mg), fluvastatin (40 mg), simvastatin (at doses of 20 to 40 mg), atorvastatin (at doses of 10 or 20 mg), pitavastatin (at doses of 2 to 4 mg), and rosuvastatin (at doses of 5 to 20 mg), typically reduce LDL-C levels by 30% to 50%. Low-intensity statins, including lovastatin (20 mg), pravastatin (at doses of 10 to 20 mg), simvastatin (20 mg), fluvastatin (at doses of 20 to 40 mg), and pitavastatin (1 mg), are expected to lower LDL-C levels by less than 30% [4,101,102,103]. A recent study on comparative effectiveness of atorvastatin and rosuvastatin in acute MI patients with elevated and normal liver enzymes, conducted by Chen X et al. [104], reported a higher risk of 1-year all-cause mortality for atorvastatin compared with rosuvastatin in patients with elevated liver enzymes, but no differences in those with normal liver enzymes.

Despite the good tolerability of statins, they were associated with several adverse effects that may affect adherence to statin therapy. The most significant side effects are muscle-related and include myalgia, myopathy, and rhabdomyolysis. Rhabdomyolysis is the most severe form of statin-induced muscle damage, characterized by muscle necrosis, elevated creatine kinase levels, myoglobinuria, and renal impairment. The incidence of statin-associated musculoskeletal symptoms (SAMS) ranges from 10% to 25% in patients taking statins [3,105]. Although serious side effects occur infrequently (0.1 to 8.4 per 100,000 patient-years), they can be life-threatening, making early detection and immediate intervention essential (Table 6). Other reported issues include liver (routine liver function tests—ALT, AST—being often recommended during treatment) and renal impairment, eye conditions such as cataracts, as well as bone and neurocognitive adverse events [4,69,94,95,102,106]. Another statin-related adverse effect is the risk of new onset diabetes mellitus, which, together with cataract surgery, were reported to be higher with rosuvastatin compared with atorvastatin in a recent trial [107]. Hydrophilic statins have shown a superior safety profile to lipophilic ones regarding the metabolic effects on muscles and bone mineral density in females [98,106].

The most recent 2023 algorithm for managing statin intolerance—known as SLAP (Switch, Lower dose, Alternate dosing, Polypharmacy)—provides a structured, four-step approach to reintroducing statin therapy. Switch refers to changing from lipophilic statins (e.g., simvastatin, atorvastatin, fluvastatin, lovastatin) to hydrophilic ones (e.g., pravastatin or rosuvastatin), or vice versa. Lower dose emphasizes that SAMS are dose-dependent, so reducing the dose may alleviate symptoms. Alternate dosing involves administering long half-life statins every other day to improve tolerability. Polypharmacy addresses cases where patients have partial or complete statin intolerance due to liver/kidney dysfunction or multiple medications; in such cases, combining low-dose statins with non-statin lipid-lowering agents (e.g., ezetimibe, PCSK9 inhibitors, bempedoic acid, or nutraceuticals) is recommended to achieve LDL-C targets. The SLAP strategy is part of the “rechallenge” phase, considered after a 4–6 week statin pause (“dechallenge”) due to either intolerable muscle symptoms (with or without elevated CK) or elevated muscle injury markers (CK > 4 × ULN). Using this approach, up to 70% of previously statin-intolerant patients can successfully resume therapy [111].

Statin therapy, while widely used and effective, requires careful consideration in specific populations. Traditionally contraindicated during pregnancy due to theoretical teratogenic risks and the critical role of cholesterol in fetal development, statins have recently undergone a reassessment. The U.S. FDA (Food and Drug Administration) has removed the blanket Category X warning, suggesting that in high-risk individuals—such as those with FH or established ASCVD—continuation of statins during pregnancy may be justified following individualized risk–benefit analysis [112,113]. Nonetheless, statins remain contraindicated during breastfeeding and should be avoided in most pregnancies. In pediatric populations, statin use is generally reserved for children with genetic lipid disorders like homozygous or heterozygous FH, where treatment may begin as early as 8–10 years of age, depending on the specific agent and patient profile. In elderly patients, especially those over 75 years, the risk–benefit balance becomes increasingly nuanced. Age-related pharmacokinetic changes, polypharmacy, comorbidities, and frailty all impact both efficacy and tolerability. While statins remain beneficial in secondary prevention, evidence supporting their use for primary prevention in the very old is limited and should be guided by individualized cardiovascular risk assessment and patient preferences [114,115].

The optimal timing for statin administration depends on the specific statin used and its pharmacokinetics. Statins with shorter half-lives—such as simvastatin, lovastatin, and fluvastatin—are most effective when taken in the evening, as hepatic cholesterol synthesis peaks overnight. Administering these statins at bedtime maximizes their LDL-cholesterol–lowering effect. In contrast, statins with longer half-lives—such as atorvastatin, rosuvastatin, and pitavastatin—have more sustained activity and can be taken at any time of day, with or without food. Regardless of timing, statins should be taken consistently at the same time each day to maintain steady plasma concentrations. Patient adherence and lifestyle compatibility are also important factors in determining the best time for dosing [116].

Although clinicians have an extensive therapeutic arsenal provided in clinical practice guidelines for the prevention and treatment of CVDs, inter-individual variability in the effectiveness and safety of drugs complicates the choice of optimal pharmacotherapy. The efficacy and safety of drugs can differ significantly among patients due to various factors, including genetic differences. This genetic variability adds complexity to the process of choosing the optimal therapy. Both pharmacokinetic characteristics of drugs—such as absorption, distribution, metabolism, and elimination—and pharmacodynamics—how drugs interact with target receptors and enzymes to produce therapeutic or adverse effects—are influenced by genetic factors [117,118]. Understanding how genetic variations affect drug safety and efficacy can help minimize adverse events and enhance patient care by allowing for the optimization of drug dosages and treatment outcomes. Incorporating individual pharmacogenetic information together with other patient-specific data can lead to the selection of the most appropriate therapeutic strategy, ultimately improving treatment effectiveness [117].

The organic anion-transporting polypeptide OATP1B1, which is encoded by the polymorphic gene SLCO1B1, plays a crucial role in the pharmacological effects of statins by facilitating their uptake in the liver. Genetic variations that affect the transport of statins from the bloodstream to the liver and their subsequent metabolism can influence the concentration of these drugs in the blood. Such genetic variations have been linked to changes in the pharmacokinetics of statins [117,119,120]. The genetic polymorphism of the SLCO1B1 gene reduces transporter activity, which causes an increase in systemic exposure to statins and a decrease in their concentration in liver cells, resulting in a decrease in lipid-lowering efficacy and an increase in the risk of side effects such as SAMS. The polymorphism in the SLCO1B1 gene associated with the risk of myopathy is c.521 T > C, which is present in two alleles (*5 and *15). Statins whose pharmacokinetics are affected by variants in the SLCO1B1 gene include simvastatin (the most affected), atorvastatin, pitavastatin, and to a lesser extent rosuvastatin and pravastatin [119,121,122]. The Dutch Pharmacogenetics Working Group (DPWG) and the Clinical Pharmacogenetics Implementation Consortium (CPIC) are two expert panels that provide recommendations for drug dosing based on pharmacogenetic information. These guidelines include specific recommendations for the type and dosage of statins for patients who have impaired function of protein transporters (Table S1) [3,123].

Statins are a class of medications that have been the subject of extensive pharmacogenetic studies. Simvastatin is listed in the FDA’s table of pharmacogenetic associations, which highlights its potential impact on safety and efficacy for individuals with intermediate or poor function in the SLCO1B1 transporter gene. In these subgroups, drug-gene interactions may increase the risk of adverse reactions. Additionally, atorvastatin and rosuvastatin are also included in the FDA table, indicating their potential influence on pharmacokinetic properties for patients with poor-function SLCO1B1 transporters. In these cases, drug-gene interactions can lead to higher systemic concentrations of the medications [124].

Genetic variability in the ABCG2 gene, which encodes the breast cancer-resistant protein (BCRP), is associated with a reduction in protein activity. This protein is an adenosine triphosphate-binding cassette (ABC) transporter that facilitates the efflux of compounds from cells. Reduced activity of ABCG2 can lead to elevated intrahepatic levels of statins, such as rosuvastatin, fluvastatin, and atorvastatin, resulting in greater LDL cholesterol reduction, but also a decreased hepatic excretion and an increased risk of developing myopathy and hepatic adverse effects. According to the 2022 CPIC guidelines, individuals with poor ABCG2 function should start therapy with a dose of 20 mg rosuvastatin or less. If a dose greater than 20 mg is necessary for therapeutic effectiveness, it is recommended to consider an alternative statin or combination therapy, such as a statin combined with ezetimibe (Table S1) [15,120,123,125,126].

There has been a significant association reported between genetic polymorphisms in CYP450 metabolic enzymes, specifically CYP3A4/5 and CYP2C9, and the pharmacokinetics and pharmacodynamics of statins [127]. CYP3A4 primarily metabolizes simvastatin and lovastatin (both being prodrugs), while atorvastatin is metabolized by CYP3A4 to a lesser extent. Fluvastatin, on the other hand, undergoes hepatic metabolism through the polymorphic enzyme CYP2C9 [94]. Individuals carrying the CYP3A4*22 variant and the CYP3A5*3 variant have shown increased bioavailability of simvastatin compared to those with the wild-type allele. However, it is important to note that CYP3A5 plays a less significant role in the hepatic metabolism of statins than CYP3A4 [120]. Variations in CYP2C9 can affect how patients respond to fluvastatin therapy. The CYP2C9*2 and CYP2C9*3 variants are the most extensively studied and lead to a reduction in CYP2C9 function by 30–40% and 80%, respectively. This can result in higher systemic exposure to fluvastatin, increasing the risk of SAMS. According to the 2022 CPIC guidelines, individuals classified as intermediate metabolizers of CYP2C9 should not exceed a dose of 40 mg of fluvastatin, while poor metabolizers should avoid doses greater than 20 mg. Therefore, it is essential to adjust doses accordingly in function of the patient’s genetic profile and their metabolic capacity (Table S1) [123].

Extensive use of statins alongside polypharmacy in older patients is of significant importance due to the potential statin interactions with other drugs, impacting their safety and effectiveness. Statins undergoing hepatic metabolism via CYP450 isoenzymes are susceptible to drug interactions, especially with other substrates of these CYPs. Pharmacological substrates that inhibit CYP450 may elevate the concentration of statin in the bloodstream, increasing the risk of myopathy. CYP450 inducers can reduce the statin levels in the blood, impacting their effectiveness [69,94]. Moreover, the substrates of OATP1B and BCRP can alter statin pharmacokinetics due to transporter-mediated statin-drug interactions, potentially leading to an increased risk of adverse reactions. In their recent study, Deng F et al. [126] reported that oral anticoagulant dabigatran, a BCRP inhibitor, may increase the area under the curve (AUC) of rosuvastatin by around 2-fold, and miconazole (an antifungal agent) may elicit an 80% in the AUC of rosuvastatin. Selected inducers and inhibitors of CYP3A4, CYP2C9, and inhibitors of OATP1B1 and BCRP are listed in Table 7 [69,94,126,128,129,130]. In a recent study, Morris et al. [101] identified potential statin-drug interactions that may elevate the risk of developing rhabdomyolysis as a consequence of delayed statin elimination, hypokalemic conditions, and impaired muscle function. Drug candidates include furosemide, clopidogrel, allopurinol, pantoprazole, and nitroglycerin [101]. Statins interactions involve not only drugs but also herbal foodstuffs. Concomitant administration of green tea extract and atorvastatin is reported to decrease the serum concentrations of statins significantly [131]. Grapefruit juice, due to its furanocoumarins with inhibitory effect on CYP3A4 enzymes [129], can increase the plasma levels of statins undergoing metabolism via this enzymatic system when taken together.

#### 6.2.2. Ezetimibe

Ezetimibe is a medication that modulates intestinal cholesterol absorption by inhibiting the Niemann-Pick C1-like 1 (NPC1L1) sterol transporter, which is found in the epithelial cells of the intestine. After ingestion, ezetimibe undergoes metabolic transformation in the intestines to form its phenolic glucuronide. This compound is then taken up by the liver, excreted into the bile, and subsequently reenters the intestinal lumen, where it continues to inhibit cholesterol absorption [15,120,132]. Ezetimibe can be used alone (monotherapy) or more commonly in combination with other medications such as statins or bempedoic acid. It has good oral bioavailability, and studies suggest it is better absorbed in older patients. Ezetimibe has been shown to reduce LDL-C levels by approximately 20%. By decreasing the cholesterol supply to the liver, it enhances the liver’s production of LDLR. Ezetimibe is frequently prescribed as an adjunct therapy for patients who do not achieve sufficient cholesterol reduction with statins alone or for those who are unable to tolerate statin medications. Additionally, it offers the co-benefit of improving glycemic control in diabetic patients [4,102,133,134].

Concerning the adverse effects of ezetimibe, the evidence from a review of international safety data suggested a causal association between ezetimibe monotherapy and drug-induced liver injury. Moreover, the review also suggested a risk of ezetimibe-related severe cutaneous adverse reactions (including Stevens–Johnson syndrome and toxic epidermal necrolysis) and drug reaction with eosinophilia and systemic symptoms (DRESS) syndrome [135].

Ezetimibe is generally well tolerated, yet its use requires particular attention in specific patient populations. It is contraindicated in individuals with known hypersensitivity to the drug or in cases of moderate to severe hepatic impairment, due to limited data on hepatic metabolism and potential accumulation risks [136]. In pregnancy, ezetimibe is not routinely recommended due to insufficient safety data in humans. Although some case series and animal studies suggest potential risks, it may be considered in highly selected cases of severe FH when the potential maternal cardiovascular benefit outweighs fetal risk [137]. Regarding pediatric use, ezetimibe has demonstrated efficacy and safety in children as young as 6 years with heterozygous familial or nonfamilial hypercholesterolemia, significantly reducing LDL-C without serious adverse effects, making it a viable adjunct or alternative when statins are inadequate or not tolerated [136]. In the elderly, studies such as the EWTOPIA 75 trial and SaveSAMS trial indicate that ezetimibe is both safe and effective as monotherapy or in combination with statins. It has shown meaningful reductions in cardiovascular events in patients aged ≥75 years, both for primary and secondary prevention [138]. Therefore, while ezetimibe is broadly applicable, clinicians should exercise individualized judgment when prescribing it to pregnant women, pediatric patients, or the very elderly.

Unlike statins, ezetimibe’s efficacy is not time-dependent, as its mechanism of action (a cholesterol absorption inhibitor that reduces intestinal uptake of dietary and biliary cholesterol) is not linked to the circadian rhythm of hepatic cholesterol synthesis. Therefore, ezetimibe can be administered at any time of the day, with or without food, based on patient convenience and adherence. Its long half-life (~22 h) and enterohepatic recirculation support once-daily dosing regardless of timing. When used in combination with statins, ezetimibe may be taken simultaneously or at a separate time, as no clinically significant interaction alters its absorption or effect. Consistent daily use, rather than timing, is the key to its optimal efficacy [139].

Studies have documented the impact of therapeutic regimens comprising ezetimibe on cardiovascular outcomes. The Plaque Regression with Cholesterol Absorption Inhibitor or Synthesis Inhibitor Evaluated by Intravascular Ultrasound (PRE-CISE-IVUS) study showed greater reduction in coronary atherosclerosis on serial intravascular ultrasound imaging when comparing ezetimibe supplemented with atorvastatin monotherapy [140].

The SHARP (Study of Heart and Renal Protection) showed that the association of simvastatin with ezetimibe decreased the risk of the combination of MI, coronary death, non-hemorrhagic stroke, or arterial revascularization by 17% in comparison with placebo in subjects with CKD without a cardiovascular event [7]. The Improved Reduction in Outcomes: Vytorin Efficacy International Trial (IMPROVE-IT) compared the combined simvastatin-ezetimibe, which demonstrated a modest (6.4%), although meaningful decrease in the association of cardiovascular death, MI, coronary revascularization or stroke, unstable angina requiring hospitalization, with simvastatin monotherapy in patients with an acute coronary syndrome [141]. Furthermore, the clinical benefit of these studies was linked to the degree of LDL reduction, supporting the role of ezetimibe as an essential tool for providing better lipid control in individuals at high cardiovascular risk [7].

The SHARP study (n = 9270) among subjects with CKD excluding CAD demonstrated that the combination of simvastatin with ezetimibe lowered LDL-C by 32 mg/dL and achieved a 17% decrease in major adverse cardiovascular events as compared to placebo [12]. However, multicenter EWTOPIA 75 (Ezetimibe Lipid-Lowering Trial on Prevention of ASCVD in 75 or Older; n = 3796), in Japanese older adults, ezetimibe was substantially associated with a reduction in the composite event of MI, sudden cardiac death, coronary revascularization, or stroke over a median period of 4 years (HR 0.66), consolidating the evidence of the benefits of LDL-C reduction in the primary prevention of ASCVD in subjects aged 75 years and older [138].

Ezetimibe undergoes almost complete metabolism by uridine diphosphate glucuronosyltransferases (UGT) in enterocytes, primarily mediated by UGT1A1 enzymes, as well as UGT1A3 and UGT2B15. Ezetimibe and its glucuronide form are substrates for ABCC2 (MRP2), an ATP-dependent cassette transporter involved in enterohepatic drug recirculation. Ezetimibe-glucuronide is also a substrate for OATP1B1, which mediates hepatic uptake of the drug. There is also some evidence for interaction with P-glycoprotein (P-gp), encoded by the ABCB1 gene [120,142]. The study of González-Iglesias et al. [142] evaluating the impact of genetic variation in transporter and metabolizing enzymes on ezetimibe pharmacokinetics revealed no significant impact of genetic polymorphism in metabolizing enzymes on drug pharmacokinetic parameters, but a trend toward a higher Cmax was observed in ABCC2 rs2272697 and ABCB1 rs2032582 variants. In the case of the OATP1B1 transporter, a non-significant increase in AUC_72h_ was observed in individuals with intermediate and poor function compared to those with normal function [142].

Ezetimibe has the potential to interact with other drugs, primarily through competition for transporters involved in absorption and elimination. This may be of clinical relevance, particularly in patients with reduced transporter function or genetic variations that affect drug pharmacokinetics [143].

## 7. Comparative Effectiveness and Safety of the Conventional Lipid-Lowering Therapies, and Pharmacoeconomic Considerations

Conventional lipid-lowering therapies, including statins and/or ezetimibe, have shown variable effectiveness and safety in mitigating cardiovascular risk (Table 8 and Table 9). Statins continue to serve as the foundation/the cornerstone of lipid management, supported by extensive evidence demonstrating their capacity to lower LDL-C levels and reduce cardiovascular events, especially when associated with ezetimibe. From a pharmacoeconomic standpoint, statins—particularly generic formulations—offer a highly favorable cost-effectiveness profile, balancing clinical efficacy, safety, and affordability. In contrast, the introduction of novel lipid-lowering agents has brought forth significant pharmacoeconomic challenges. Although statins and ezetimibe remain widely accessible and cost-efficient, newer treatments such as PCSK9 inhibitors, inclisiran, lomitapide, and evinacumab entail considerably higher acquisition and long-term monitoring costs. As a result, their use is typically limited to specific high-risk groups, including patients with familial hypercholesterolemia or those unable to reach LDL-C targets despite optimal conventional therapy. Cost-effectiveness analyses consistently demonstrate that these advanced therapies yield the greatest economic value when directed at individuals with the highest absolute cardiovascular risk, where the clinical benefits justify the higher expenditures. Moreover, reimbursement decisions by agencies such as NICE increasingly incorporate formal health-economic evaluations, leading to more restrictive eligibility criteria and treatment thresholds. In this context, integrating pharmacoeconomic considerations with clinical efficacy and safety is critical to supporting sustainable and equitable access to innovative lipid-lowering treatments [17,69,79,144,145,146,147,148,149].

## 8. Conclusions

Hypercholesterolemia remains a central driver in the pathogenesis of atherosclerotic cardiovascular disease and related complications, requiring ongoing and personalized management strategies. Conventional lipid-lowering therapies, particularly statins and ezetimibe, continue to play a vital role in reducing LDL-C and cardiovascular risk, especially when guided by evidence-based risk assessment tools and international clinical guidelines. However, treatment gaps persist, notably in patients with familial hypercholesterolemia or very high cardiovascular risk who do not achieve lipid targets with standard therapy alone. Addressing these gaps demands a multifaceted approach that includes lifestyle modification, pharmacogenomic insights, and pharmacoeconomic considerations to ensure both efficacy and long-term adherence. While this first part of our review has focused on the pathophysiology of dyslipidemia and the clinical application of conventional therapies, Part II will present an in-depth analysis of emerging lipid-lowering agents—including PCSK9 inhibitors, inclisiran, lomitapide, bempedoic acid, and evinacumab—their clinical effectiveness, safety profiles, and implications for future treatment algorithms. Together, these two complementary reviews aim to provide a complete, up-to-date resource for optimizing dyslipidemia management in contemporary cardiovascular care.

## Figures and Tables

**Table 1:** Key clinical and genetic differences between heterozygous and homozygous FH [36].

Characteristic	Heterozygous FH (HeFH)	Homozygous FH (HoFH)
**Prevalence**	~1 in 250 individuals	~1 in 300,000 individuals
**Genetic basis**	one pathogenic mutation (LDLR, ApoB, or PCSK9)	two pathogenic mutations (same gene or compound heterozygous)
**LDL-C levels**	typically 190–400 mg/dL	frequently > 500 mg/dL
**Age of onset**	early adulthood	childhood
**Clinical features**	tendon xanthomas, premature CAD	cutaneous xanthomas, arcus cornealis, aortic stenosis, early MI
**Response to statins**	generally good	minimal to poor
**Response to PCSK9 inhibitors**	effective in most cases	limited efficacy, especially in null LDLR mutations
**Need for advance therapy**	rarely required	often necessary (lipoprotein apheresis, lomitapide, evinacumab)
**Cardiovascular risk**	increased 10–20× vs. general population	extremely high; events often before age 20 if untreated
**Prognosis without treatment**	events typically after age 30–40	death or major cardiovascular events in adolescence/young adulthood

**Table 2:** Lipid targets according to clinical context and cardiovascular risk [69,70].

Clinical Context/Risk Category	LDL-C Target	Non-HDL-C Target
Established ASCVD (CAD, stroke, PAD)	<55 mg/dL (1.4 mmol/L)	<85 mg/dL (2.2 mmol/L)
Diabetes mellitus with target organ damage (nephropathy, retinopathy, neuropathy)	<55 mg/dL	<85 mg/dL
Severe CKD (GFR < 30 mL/min/1.73 m^2^)	<55 mg/dL	<85 mg/dL
Diabetes without organ damage	<70 mg/dL (1.8 mmol/L)	<100 mg/dL (2.6 mmol/L)
Moderate CKD (GFR 30–59 mL/min/1.73 m^2^)	<70 mg/dL	<100 mg/dL
Heterozygous FH without ASCVD	<70 mg/dL	<100 mg/dL
Heterozygous FH with ASCVD or major risk factor	<55 mg/dL	<85 mg/dL
Severe hypertriglyceridemia (>500 mg/dL)	LDL-C per CV risk	Non-HDL-C per CV risk
Cerebrovascular atherosclerotic disease (ischemic stroke)	<55 mg/dL	<85 mg/dL
Hypertension without ASCVD or diabetes	LDL-C per global risk	Non-HDL-C per global risk
Moderate global cardiovascular risk	<100 mg/dL (2.6 mmol/L)	<130 mg/dL (3.4 mmol/L)
Low global cardiovascular risk	<116 mg/dL (3.0 mmol/L)	<145 mg/dL (3.8 mmol/L)

Legend: ASCVD—atherosclerotic cardiovascular disease; CAD—coronary artery disease; PAD—peripheral artery disease; CKD—chronic kidney disease; FH—familial hypercholesterolemia; CV risk—cardiovascular risk stratification.

**Table 3:** Comparative overview of SCORE2 and PCE in cardiovascular risk assessment [74].

Criteria	SCORE2 (ESC)	PCE (ACC/AHA)
**Geographic/Population focus**	Europe (calibrated for low-, moderate-, high-, very-high risk countries)	United States population (including adjustments for Black vs. White individuals)
**Age group**	40–69 years (SCORE2), extended to 70+ with SCORE2-OP	40–79 years
**Type of risk estimated**	10-year risk of first fatal and non-fatal CVD events (MI, stroke)	10-year risk of first ASCVD event (MI, stroke, cardiovascular death)
**Variables included**	Age, sex, smoking status, systolic blood pressure, total cholesterol, or LDL-C	Age, sex, race, total cholesterol, HDL-C, systolic BP, diabetes, smoking, blood pressure treatment
**Output format**	Percentage (%) risk; categorized into 4 levels—low, moderate, high, very high	Percentage (%) risk; categorized into 4 levels -low, borderline, intermediate, high
**Risk categories**	<5%—Low	<5%—Low
	5–10%—Moderate	5–7.4%—Borderline
	10–15%—High	7.5–19.9%—Intermediate
	≥15% or clinical ASCVD—Very high	≥20%—High
**Use of modifiers**	Lifetime risk, family history, social determinants, CAC score, imaging (optional)	Risk-enhancing factors (e.g., hs-CRP, CKD, Lp(a), CAC score) for refined decision-making

**Table 4:** Comparison of ESC, ACC/AHA, and NICE guidelines for LDL-C targets and treatment strategies [74,79].

Risk Category/Patient Group	ESC/EAS (LDL-C Target)	ACC/AHA (Treatment Threshold)	NICE (Approach and Targets)
**Very high risk (e.g., ASCVD, diabetes + target organ damage, CKD)**	<55 mg/dL and ≥50% reduction	High-intensity statin ± ezetimibe/PCSK9i if LDL-C ≥ 70 mg/dL	High-intensity statin; consider ezetimibe or PCSK9i if ≥40% non-HDL-C reduction not achieved on maximal statin
**High risk**	<70 mg/dL	≥50% LDL-C reduction with high-intensity statin	High-intensity statin as first line; ezetimibe if needed
**Moderate risk**	<100 mg/dL	Moderate-intensity statin personalized based on risk enhancers	Offer statin if ≥10% 10-year risk; aim for ≥40% non-HDL-C reduction
**Low risk**	<116 mg/dL	Lifestyle modification statin optional based on discussion	Lifestyle modification; statin only if significant risk factors present
**Severe hypercholesterolemia (LDL-C ≥ 190 mg/dL)**	Treat as high or very high risk	High-intensity statin add ezetimibe/PCSK9i if needed	High-intensity statin; consider ezetimibe; PCSK9i reserved for familial hypercholesterolemia meeting strict criteria
**Diabetes mellitus (age 40–75)**	Risk-based LDL-C targets	At least moderate-intensity statin high-intensity if multiple risks	High-intensity statin if QRISK ≥ 10% or evidence of end-organ damage

**Table 5:** Dietary sources of trans fatty acids, polyunsaturated fatty acids, and phytosterols [90,91,92].

Trans Fatty Acids	Polyunsaturated Fatty Acids	Phytosterols
margarine	corn oil	seeds
crackers	soybean oil	fruits
animal products	sunflower oil	legumes
cakes	walnuts	nuts
potato chips	sunflower seeds	vegetables
lard	tofu	vegetable oils
biscuits	soybeans	vegetable oil-based margarines
popcorn		cereal grains

Note: The leading dietary sources of trans fats are margarine, crackers, animal products, cakes, potato chips, lard, biscuits, and popcorn [90]. Important sources of polyunsaturated fatty acids include corn oil, soybean oil, sunflower oil, some nuts and seeds such as walnuts and sunflower seeds, tofu, and soybeans [91]. Phytosterols can be naturally identified in all plant-based foods and are detected in seeds (i.e., sesame, sunflower, flax, chia, pumpkin seeds), fruits (i.e., avocados, oranges, berries, bananas, apples), legumes (i.e., lentils, chickpeas, soybeans, black beans, kidney beans), nuts (i.e., pistachios, almonds, walnuts, pecans, cashews, hazelnuts), vegetables (i.e., brussels sprouts, broccoli, cauliflower, carrots, spinach, kale), vegetable oils (i.e., wheat germ, corn, canola, soybean, sunflower, olive oil), vegetable oil-based margarines (fortified foods), and cereal grains (i.e., whole-wheat bread, flour, wheat bran, brown rice, oats, barley, cornmeal) [92].

**Table 6:** Criteria for statin discontinuation according to guidelines [17,69,70,79].

Guideline	Parameter	Threshold for Discontinuation
ESC/EAS	Transaminases (ALT/AST)	>3 × Upper Limit of Normal (ULN), confirmed on repeat testing
	Creatine Kinase (CK)	>10 × ULN (with or without symptoms) or >5 × ULN with significant muscle symptoms
AHA/ACC	Transaminases (ALT/AST)	Persistent elevation >3 × ULN
	Creatine Kinase (CK)	>10 × ULN or >5 × ULN with muscle symptoms
NICE	Transaminases (ALT/AST)	ALT >3 × ULN on repeat testing
	Creatine Kinase (CK)	>10 × ULN regardless of symptoms, or >5 × ULN with muscle pain
KDIGO	Transaminases (ALT/AST)	>3 × ULN
	Creatine Kinase (CK)	>10 × ULN or symptomatic elevations

Legend: ALT—Alanine Aminotransferase; AST—Aspartate Aminotransferase; ULN—Upper Limit of Normal; KDIGO—Kidney Disease: Improving Global Outcomes. Note: 1. Liver function tests—Current clinical guidelines recommend baseline liver function testing before initiating statin therapy, but routine periodic monitoring is generally not required in asymptomatic patients. The ACC/AHA advises measuring ALT prior to starting treatment and only repeating tests if symptoms of hepatotoxicity arise, such as fatigue, jaundice, or dark urine. Similarly, the ESC/EAS recommends baseline ALT measurement and further testing only if liver dysfunction is suspected. In contrast, the UK’s National Institute for Health and Care Excellence (NICE) suggests checking transaminases at baseline, 3 months, and 12 months, with no need for ongoing routine monitoring unless ALT exceeds three times the upper limit of normal or clinical concerns persist. Across all guidelines, discontinuation of statin therapy is typically considered only when ALT is elevated beyond three times the upper limit of normal or if liver-related symptoms are present [17,69,79,108,109]. 2. Muscle-related side effects—Clinicians should assess for muscle symptoms at baseline and during follow-up, especially in high-risk patients such as older adults, those with hypothyroidism, or those taking interacting medications. If a patient reports muscle symptoms, CK levels should be measured. If CK is mildly elevated (less than 3–5 times the upper limit of normal), statins may often be continued with monitoring. If CK exceeds 10 times the upper limit or if rhabdomyolysis is suspected, statin therapy should be discontinued immediately, and renal function assessed. Re-challenging with a lower dose or an alternative hydrophilic statin (e.g., pravastatin or rosuvastatin) may be considered once symptoms resolve. Alternate-day dosing or non-statin lipid-lowering therapies may also be appropriate for patients with persistent statin intolerance [110,111].

**Table 7:** Selected inducers and inhibitors of CYP3A4, CYP2C9, and inhibitors of OATP1B1 and BCRP [69,94,126,128,129,130].

Isoenzyme/Transportor	CYP3A4	CYP2C9	OATP1B1	BCRP
**Statins**	Atorvastatin, lovastatin, simvastatin	Fluvastatin, rosuvastatin	Atorvastatin, fluvastatin, lovastatin, pitavastatin, pravastatin, rosuvastatin, simvastatin,	Rosuvastatin, fluvastatin, atorvastatin
**Inhibitors**	Amiodarone, amlodipine, clarithromycin, cyclosporine A, danazol, diltiazem, erythromycin, fluconazole, fluoxetine, fluvoxamine, gemfibrozil, grapefruit juice, isoniazid, itraconazole, ketoconazole, mibefradil, midazolam, nefazodone, posaconazole, protease inhibitors, ranolazine, sertraline, tacrolimus, telithromycin, ticagrelor, tricyclic antidepressants, verapamil	Amiodarone, capecitabine, fluconazole, fluvoxamine, ketoconazole, metronidazole, miconazole, sulfamethoxazole + trimethoprim, voriconazole, zafirlukast	Clarithromicine, coumestrol, cyclosporine, diazepam, diethylstilbestrol, erythromycin, erlotinib, estrone-3-sulfate, gemfibrozil, genistein, ivermectin, ketoconazole, novobiocin, rifampicin, ritonavir, roxithromycin, sacubitril, telithromycin, tipranavir, verapamil, vemurafenib	Cyclosporine, dabigatran etexilate, elacridar, everolimus, ketoconazole, meloxicam, miconazole, rifampicin, tyrosine-kinase inhibitors (sunitinib, gefitinib, imatinib), vemurafenib, ziprasidone,
**Inducers**	Aprepitant, carbamazepine, cyclophosphamide, corticosteroids, efavirenz, nevirapine, phenytoin, pioglitazone, phenobarbital, St. John’s wort	Carbamazepine, phenobarbital, phenytoin, rifampin		

**Table 8:** Comparative effectiveness and safety of statins and ezetimibe [69,141,150,151,152,153,154,155,156].

Parameter	Statins	Ezetimibe
**Average LDL-C Reduction**	30–60%	~20% in monotherapy; additional 21–27% when added to statin (vs. placebo)
**HDL-C Increase**	10–30%	3–5%
**MACE Reduction (vs. placebo)**	18% overall risk reduction (secondary prevention) >20% (primary prevention)	Decrease MACE risk when added to statins (e.g., IMPROVE-IT)
**Effect on All-Cause Mortality**	Significant reduction, especially in high-risk patients 65% reduction	No significant reduction alone; some benefit when combined
**Myopathy Risk**	10–25%	Not significantly increased over placebo
**Liver Enzyme Elevation**	Borderline elevation of liver function tests (LFTs), more commonly with potent statins or high doses	Relatively rare Improves serum ALT levels
**New-Onset Diabetes Risk**	Slightly increased, especially with high-intensity statins	Improves insulin resistance
**Other Common Side Effects**	GI system disorders,	GI system disorders, arthralgia, headache
**Use in Statin Intolerance**	Limited (lower dose or alternate statin often tried)	Preferred non-statin option

Legend: MACE—Major Adverse Cardiovascular Events; LFTs—liver function tests; GI—gastrointestinal; ALT—Alanine aminotransferase.

**Table 9:** Comparative assessment of the efficacy and safety profile of statin monotherapy and statin plus ezetimibe combination [141,150,151,154,155,156,157,158,159,160,161,162,163,164].

Parameter	Statin Monotherapy	Statin + Ezetimibe Combination
**Average LDL-C Reduction**	30–60%	An additional 23–24%, on average
**HDL-C Increase**	10–30%	No significant differences
**Additional LDL-C drop vs. doubling statin dose**	-	Combination produces greater LDL-C drop than doubling statin dose
**MACE Reduction (vs. placebo)**	18% overall risk reduction (secondary prevention) >20% (primary prevention)	18% reduction (odd ratio [OR], 0.82) vs. statin alone in meta-analysis of 108,373 patients
**All-cause mortality**	Significantly reduced risk	Superior to statin monotherapy; 19% reduction (OR, 0.81) vs. statin monotherapy
**Stroke reduction**	Included in MACE outcomes	17% reduction (OR, 0.83)
**Time to prevent 1 MACE/100 patients**	19.6 months of intensive statin therapy	~ 36 months
**Adverse events/discontinuation**	Mild muscle pain; elevated LFTs in ~1–3% of patients (<2 × the upper limit of normal); new-onset diabetes risk increased	Comparable AE profile to statin alone in meta-analysis; combination had fewer discontinuations in elderly/diabetics Lower risks of discontinuation
**Myalgia/myopathy**	10–25%	Significant reduction in muscle-related AE for Low/Moderate-intensity Statins and Ezetimibe
**Liver enzymes (ALT/AST)**	Usually transient elevation of transaminase	Favorable to statin monotherapy No significant difference in ALT levels, greater increase in AST levels vs. high-intensity statin monotherapy
**New-onset diabetes**	Slightly increased risk with high-intensity statins	Reduced events of new-onset diabetes for low/moderate-intensity statins and ezetimibe No increase in the risk of new-onset diabetes when adding ezetimibe to statin therapy
**GI, headache, others**	Statin: GI symptoms, headache, fatigue; Ezetimibe: mild GI, URTI, arthralgia	Combined profile acceptable; no increase in overall adverse events

Legend: MACE—Major Adverse Cardiovascular Events; AE—adverse events; LFTs—liver function tests; ALT—Alanine Aminotransferase; AST—Aspartate Aminotransferase; GI—gastrointestinal.

## Data Availability

There are no additional data to be published.

## Supplementary Materials

The following supporting information can be downloaded at: https://www.mdpi.com/article/10.3390/life15081185/s1, Table S1: The DPWG and CPIC therapeutic recommendations for statins.

## Abbreviations

The following abbreviations are used in this manuscript:

ABC	Adenosine triphosphate-binding cassette
ABCA1	ATP-binding cassette transporter A1
ACC	American College of Cardiology
ACL	Adenosin triphosphate-citrate lyase
AHA	American Heart Association
ALT	Alanine Aminotransferase
ApoB	Apolipoprotein B
ASCVD	Atherosclerotic cardiovascular disease
AST	Aspartate Aminotransferase
AUC	Area under the curve
BCRP	Breast cancer-resistant protein
BMI	Body mass index
BP	Blood pressure
CAC	Coronary artery calcium
CAD	Coronary artery disease
CK	Creatine Kinase
CKD	Chronic kidney disease
COPD	Chronic Obstructive Pulmonary Disease
CPIC	Clinical Pharmacogenetics Implementation Consortium
CVD	Cardiovascular disease
CYP450	Cytochrome P450
DAST	Dietary Approaches to Stop Hypertension
DPWG	Dutch Pharmacogenetics Working Group
DRESS	Drug reaction with eosinophilia and systemic symptoms
ESC	European Society of Cardiology
FDA	Food and Drug Administration
FH	Familial hypercholesterolemia
FHS	Framingham Heart Study
HDL	High-density lipoprotein
HDL-C	High-density lipoprotein cholesterol
HeFH	Heterozygous familial hypercholesterolemia
HMG-CoA	β-hydroxy β-methylglutaryl-coenzyme A
HoFH	Homozygous familial hypercholesterolemia
IBD	Inflammatory Bowel Disease
IDL	Intermediate-density lipoproteins
IMPROVE-IT	Improved Reduction in Outcomes: Vytorin Efficacy International Trial
KDIGO	Kidney Disease: Improving Global Outcomes
LDL	Low-density lipoprotein
LDL-C	Low-density lipoprotein cholesterol
LDLR	Low-density lipoprotein receptor
LDLRAP	Low-density lipoprotein receptor adaptor protein
LFTs	Liver function tests
LPL	Lipoprotein lipase
MACE	Major Adverse Cardiovascular Events
MI	Myocardial infarction
MTP	Microsomal triglyceride transfer protein
NICE	National Institute for Health and Care Excellence
NPC1L1	Niemann-Pick C1-like 1
OATP	Organic anion-transporting polypeptide
oxLDL	Oxidized low-density lipoprotein
PAD	Peripheral arterial disease
PCE	Pooled Cohort Equations
PCOS	Polycystic ovary syndrome
PRE-CISE-IVUS	Plaque Regression with Cholesterol Absorption Inhibitor or Synthesis Inhibitor Evaluated by Intravascular Ultrasound
PUFAs	Polyunsaturated fats
SAMS	Statin-associated musculoskeletal symptoms
SHARPP	Study of Heart and Renal Protection
SR-BI	Scavenger receptor class B type I
TG	Triglycerides
UGT	Uridine diphosphate glucuronosyltransferases
ULN	Upper Limit of Normal
VLDL	Very-low-density lipoprotein
