# Supplementary material for: A Comprehensive Review of the Latest Approaches to Managing Hypercholesterolemia: A Comparative Analysis of Conventional and Novel Treatments: Part I
Source: Life (Basel). 2025 Jul 25;15(8):1185. doi: 10.3390/life15081185 (PMC12387733; doi:10.3390/life15081185)
Supplement: Supplementary file 1 [file life-15-01185-s001.zip › life-3730296-supplementary.pdf]

# A Comprehensive Review of the Latest Approaches to Managing Hypercholesterolemia: A Comparative Analysis of Conventional and Novel Treatments: Part I

**Ema-Teodora Nițu [1,2,3§], Narcisa Jianu [1,3§], Cristina Merlan[1,3], Darius Foica [3], Laura Sbârcea [1,2*], Valentina Buda [1,4], Maria Suciu [1,4], Adelina Lombrea [1], Dana Emilia Movilă [5,6]**

[1] Faculty of Pharmacy, "Victor Babeș" University of Medicine and Pharmacy, 2 Eftimie Murgu Square, 300041 Timișoara, Romania; ema-teodora.nitu@umft.ro (E-T.N.); narcisa.dinu@umft.ro (N.J.); sbarcea.laura@umft.ro (L.S.); buda.valentina@umft.ro (V.B.); suciu.maria@umft.ro (M.S.); adelina.lombrea@umft.ro (A.L.)
[2] Advanced Instrumental Screening Center, Faculty of Pharmacy, Victor Babeş University of Medicine and Pharmacy, 2 Eftimie Murgu Square, 300041 Timisoara, Romania; ema-teodora.nitu@umft.ro (E-T.N.); sbarcea.laura@umft.ro (L.S.)
[3] Doctoral School, "Victor Babeş" University of Medicine and Pharmacy, 2 Eftimie Murgu Street, 300041 Timisoara, Romania; ema-teodora.nitu@umft.ro (E-T.N.); narcisa.dinu@umft.ro (N.J.); darius.foica@umft.ro (D.F.); cristina.merlan@umft.ro (C.M.)
[4] Research Centre for Pharmaco-Toxicological Evaluation, "Victor Babeș" University of Medicine and Pharmacy, 2 Eftimie Murgu Square, 300041 Timisoara, Romania; buda.valentina@umft.ro (V.B.); suciu.maria@umft.ro (M.S.)
[5] University Clinic of Internal Medicine and Ambulatory Care, Prevention and Cardiovascular Recovery, Department VI—Cardiology, "Victor Babes" University of Medicine and Pharmacy, 3000041 Timisoara, Romania; man.dana@umft.ro (D-E.M)
[6] Research Centre of Timisoara Institute of Cardiovascular Diseases, "Victor Babes" University of Medicine and Pharmacy, 3000041 Timisoara, Romania; man.dana@umft.ro (D-E.M)
* Correspondence: sbarcea.laura@umft.ro (L.S.)
§ These authors contributed equally to this review article

## Supplementary materials

**Table S1.** The DPWG and CPIC therapeutic recommendations for statins [3, 123]

| Drug | Transportor/ Enzyme | Phenotype | Classification of recommendation (CPIC)/ (DPWG) | CPIC Therapeutic recommendations | DPWG Therapeutic recommendations |
|---|---|---|---|---|---|
| Atorvastatin | SLCO1B1 | poor function | Moderate/ Potentially beneficial | Prescribe ≤ 20 mg as a starting dose; adjust doses based on disease-specific guidelines. If doses > 20 mg are required for desired efficacy, consider rosuvastatin or combination therapy (atorvastatin | Adjustment on atorvastatin therapy in patients with additional risk factors for statin-induced myopathy<br>a) Choose an alternative statin less affected by gene variation |

| | | | | | |
|---|---|---|---|---|---|
| | | | | plus nonstatin guideline-directed therapy) | Rosuvastatin and pravastatin are similarly influenced by the SLCO1B1 genetic variation, but are not affected by CYP3A4 inhibitors (amiodarone, verapamil and diltiazem). Fluvastatin is not significantly affected by the SLCO1B1 gene variation or CYP3A4 inhibitors.<br>b) If an alternative is not an option, keep the required dose as less as possible (by adding ezetimibe) |
| Rosuvastatin | SLCO1B1 | poor function | Moderate/<br>Potentially beneficial | Prescribe ≤ 20 mg as a starting dose; adjust doses based on disease-specific guidelines. If doses > 20 mg are required for desired efficacy, combination therapy should be considered (rosuvastatin plus nonstatin guideline-directed therapy) | In patient with additional risk factors for statin-induced myopathy, keeping the required dose as less as possible is recommended (by adding ezetimibe) |
| Simvastatin | SLCO1B1 | poor function | Strong/<br>Essential (80mg/day)<br>Beneficial (40 mg/day) | Prescribe an alternative statin depending on the desired potency | Choosing an alternative is recommended<br>Consider any additional factors for statin-induced myopathy<br>Atorvastatin is influenced to a lesser extent by the SLCO1B1 gene variation, but is affected by CYP3A4 inhibitors, including amiodarone, verapamil and diltiazem. Atorvastatin in not recommended to be used for patients with additional risk factors for statin-induced myopathy.<br>Rosuvastatin and pravastatin are less affected the SLCO1B1 gene variation and are not affected by CYP3A4 inhibitors.<br>Fluvastatin is not significantly affected by the SLCO1B1 gene variation or CYP3A4 inhibitors. |

| | | | | | |
|---|---|---|---|---|---|
| | | | | | If an alternative in not an option avoid simvastatin doses > 40 mg/day (by adding ezetimibe) |
| Fluvastatin | SLCO1B1 | poor function | Moderate/- | Prescribe ≤ 40 mg/day as a starting dose; adjust doses based on disease-specific guidelines. If 40 mg/day is tolerated, but higher potency is required, a higher dose or an alternative statin or combination therapy (fluvastatin plus nonstatin guideline-directed therapy) could be considered. Physician should be aware of possible increased risk for myopathy with fluvastatin doses > 40 mg/day | - |
| Lovastatin | SLCO1B1 | poor function | Moderate/- | Prescribe an alternative statin depending on the desired potency | - |
| Pravastatin | SLCO1B1 | poor function | Moderate/- | Prescribe ≤ 40 mg/day as a starting dose; adjust doses based on disease-specific guidelines. If 40 mg/day is tolerated, but higher potency is required, a higher dose or an alternative statin or combination therapy (pravastatin plus nonstatin guideline-directed therapy) could be considered. Physician should be aware of possible increased risk for myopathy with pravastatin doses > 40 mg/day | - |
| Pitavastatin | SLCO1B1 | poor function | Moderate/- | Prescribe ≤ 1 mg as a starting dose and adjust doses based on disease-specific guidelines. If doses exceeding 1 mg are needed for desired efficacy, an alternative statin or combination therapy | - |

| | | | | | |
|---|---|---|---|---|---|
| | | | | (pitavastatin plus nonstatin guideline-directed therapy) should be considered. | |
| Rosuvastatin | ABCG2 | poor function | Moderate/- | Prescribe ≤ 20 mg as a starting dose; adjust doses based on disease-specific and population-specific guidelines. If doses > 20 mg are required for desired efficacy, an alternative statin or combination therapy should be considered (rosuvastatin plus nonstatin guideline-directed therapy) | - |
| Fluvastatin | CYP2C9 | intermediate metabolizer | Moderate/- | Prescribe ≤ 40 mg/day as a starting dose; adjust doses based on disease-specific guidelines. If dose > 40 mg needed for desired efficacy, consider an alternative statin or combination therapy (fluvastatin plus nonstatin guideline-directed therapy) | - |
| | | poor metabolizer | Moderate/- | Prescribe ≤ 20 mg/day as a starting dose; adjust doses based on disease-specific guidelines. If dose > 20 mg needed for desired efficacy, consider an alternative statin or combination therapy (fluvastatin plus nonstatin guideline-directed therapy) | - |
